# Standardization and normative data of the Greek version of the temperament and character inventory (TCI)

**DOI:** 10.1186/s12991-015-0067-x

**Published:** 2015-09-22

**Authors:** Konstantinos N. Fountoulakis, Sandor Rozsa, Melina Siamouli, Katerina Moutou, Eleonora Pantoula, Claude Robert Cloninger

**Affiliations:** 3rd Department of Psychiatry, Aristotle University of Thessaloniki, Thessalonikis, Greece; Department of Psychiatry, Washington University School of Medicine, St. Louis, MO USA; Aristotle University of Thessaloniki, Thessalonikis, Greece

## Abstract

**Background:**

Robert Cloninger’s psychobiological model of temperament and character is a dimensional approach to personality assessment and gave birth to the temperament and character inventory (TCI). The aim of the present report is to examine the psychometric properties of the Greek version of the TCI, and to replicate its postulated structure and provide preliminary normative data for the Greek population.

**Methods:**

The study sample included 734 subjects from the general Greek population (436 females; 59.4 % and 298 males; 40.6 %). Their mean age was 40.80 ± 11.48 years (range 25–67 years). The mean age for females was 39.43 ± 10.87 years (range 25–65 years), while the mean age for males was 42.82 ± 12.06 years (range 25–67 years). Descriptive statistics tables concerning age, gender and occupational status distribution in the sample were created. The analysis included the calculation of Cronbach’s alpha, factor analysis with promax rotation and the calculation of Pearson correlation coefficients between the subscales scores. Analysis of Covariance with age as covariate and *t* test and Cohen’s *d* as post hoc tests was used to search for differences in subscales scores between males and females.

**Results:**

The overall psychometric properties of the Greek version of the TCI proved to be satisfactory, with acceptable consistencies of the subscales. The factor analysis of temperament identified four factors which together explained 58.56 % of total variance, while the factor analysis of the three-factor solution of the character explained 52.24 % of total variance. The TCI scales correlate significantly but weakly between each other and with age.

**Discussion:**

The Greek version of the TCI exhibits psychometric properties similar to its original English counterpart and to other national translations and it is suitable for use in research and clinical practice.

## Background

The temperament theory originated probably from ancient Egypt or Mesopotamia, but a similar theory was definitely formulated by the school of Cos and specifically by Polybus, a pupil and son-in-law to Hippocrates (fourth century B.C.) in his book ‘Peri physeos anthropou’ (‘On the Nature of Man’). This was further elaborated by Eristratos, Asclepiades (first century B.C.) and eventually by Galen (second century A.D.) with his treatise ‘Peri crasaion’ (‘De temperamentis’). This approach was the standard until the sixteenth century, and in India and the Muslim world it constituted the basis of Yunani or Unani medicine (after Yunan that is Iones-Greeks in eastern languages). It was Avicenna (980–1037 A.D.) who in his book ‘The Canon of Medicine’, extended this theory to include ‘emotion, mental ability, moral attitudes, self-awareness, movement and dreams’. Eventually, the temperament theory influenced philosophical thinking and played a predominant role in several theoretical and scientific areas, such as Gnosticism, Materialism, Anthropology, Idealism, Iatromechanism and Existentialism [1].

In modern era, Emil Kraepelin (1909–1915) described four basic affective dispositions: depressive, manic, irritable and cyclothymic and Alfred Adler (1879–1937) spoke of four mistaken goals: ‘recognition’, ‘power’, ‘service’, and ‘revenge’. Eduard Spränger (1882–1963) suggested four human values: ‘religious’, ‘theoretic’, ‘economic’, and ‘artistic’, while William James (1842–1910) spoke of tough-minded and tender-minded temperaments. Ernst Kretschmer (1888–1964) described the body types (asthenic/leptosomic, athletic and pyknic) and the related temperaments, with the pyknic-body type being extrovert and related to manic depression. He also divided personality into two constitutional groups (temperaments): the ‘schizothymic’ (with the hyperesthetic–sensitive and the anesthetic-cold characters) and the ‘cyclothymic’ (with the depressive–melancholic and the hypomanic characters). Hans Eysenck [2–11], Jeffrey Gray [12], Jerome Kagan [13–21] and Hagop Akiskal [22–28] all developed theories of temperament and character traits and dimensions.

Robert Cloninger’s psychobiological model of temperament and character is a dimensional approach to personality assessment. It measures both normal and abnormal personality traits in the two major components of personality, temperament and character [29–32]. It proposes the existence of four dimensions of temperament and three dimensions of character. Each reflects normally distributed quantitative traits present in varying degrees in everyone. Temperament dimensions [novelty seeking (NS), harm avoidance (HA), reward dependence (RD), and persistence (PS)] stand for styles that are moderately stable throughout life and concern automatic basic emotional responses such as anger, fear, and disgust. Character dimensions [self-directedness (SD), cooperativeness (CO), and self-transcendence (ST)], stand for individual differences in goals, values, and self-conscious emotions such as shame, guilt, and empathy, and mature in a stepwise fashion.

The aim of the present report is to examine the psychometric properties of the Greek version of the TCI, and to replicate its postulated structure and provide preliminary normative data for the Greek population.

## Methods

The study sample included 734 subjects from the general Greek population (436 females; 59.4 % and 298 males; 40.6 %). Their mean age was 40.80 ± 11.48 years (range 25–67 years). The mean age for females was 39.43 ± 10.87 years (range 25–65 years), while the mean age for males was 42.82 ± 12.06 years (range 25–67 years).

This was a convenient sample, not representative of the general population of the country but with similar sociodemographic characteristics of the mentally healthy and occupationally active population of Greece.

All subjects gave written informed consent and the study was approved by the Ethics committee of the University.

The TCI was translated into Greek by KNF and back translated into English by two other authors (MS and KM). The accuracy of the translation and its conformity with the original version were checked by the originators of the instrument and KNF. Discrepancies were discussed until an agreement was reached. This version was then refined, paying special attention to the use of frequent and well-known words and using a correct and easy grammar, to ensure that the items were well understood for every level of education.

The original English version requires a ‘true/false’ response. In the Greek language, two bipoles of words correspond to this: ‘true/lie’ and ‘correct/false’. The second bipole was chosen and used. The Greek version of the TCI can be downloaded from the Anthropedia Foundation at http://anthropedia.org.

Descriptive statistics tables concerning age, gender and occupational status distribution in the sample were created. The analysis included the calculation of Cronbach’s alpha for each of the temperaments and characters and the identification of psychometrically weak items. However, these items were retained in the final version of the scale to keep compatibility with other national versions as well as with the original one. The means and standard deviations for each subscale were calculated. The analysis also included factor analysis with promax rotation and the calculation of Pearson correlation coefficients between the subscales scores. Analysis of Covariance with age as covariate and *t* test and Cohen’s *d* as post hoc tests was used to search for differences in subscales scores between males and females.

## Results

The age and gender distribution in the study sample, compared to the general Greek population according to the 2009 census is shown in Table 1. The distribution of occupation in the study sample (data were available for 533 subjects) is shown in Table 2. It seems that the study sample, though convenient and not epidemiologically derived, is representative of the country’s active population with some overrepresentation of younger ages.

**Table 1:** Composition of the study sample in terms of gender and age in comparison to the general population according to the Greek National Statistics Service for 2009

Age group	Greek population (approximation for 2009)	Study sample
Total population	11,282,751	734
Females vs. males	48 vs. 52 %	40.6 vs 59.4 %
25–29 years old	11.02 %	25.81 %
29–34 years old	11.31 %	12.90 %
34–39 years old	10.00 %	15.44 %
40–44 years old	10.00 %	13.13 %
44–49 years old	9.21 %	10.60 %
50–54 years old	8.92 %	10.14 %
55–59 years old	6.83 %	8.29 %
60–64 years old	7.09 %	3.69 %

**Table 2:** Occupation characteristics of the study sample

	Count	%
He/she used to work but is currently unemployed	0	0.00
He/she never worked and neither does now	0	0.00
Clerk (civil or private)	338	63.41
Free professional (tradesman, craftsman)	62	11.63
Doctor, lawyer, engineer, priest, teacher, etc.	75	14.07
Student (college or university)	12	2.25
Blue collar worker (construction worker, farmer)	26	4.88
Housewife	20	3.75
Total	533	100.00

The means and standard deviations of individual subscales and the difference between males and females are shown in Table 3 along with Cronbach’s alpha values. The factor analysis results of temperament and character scales are shown separately in Tables 4 and 5. The principal component analysis of temperament identified four factors with eigenvalues greater than 1, which together explained 58.56 % of total variance. The eigenvalues were, respectively: 2.70, 1.93, 1.39, 1.00 (Table 4). The principal component analysis of the three-factor solution of the character explained 52.24 % of total variance (Table 5).

**Table 3:** Means and standard deviations of individual subscales and the difference between males and females

TCI scale/subscale	No. of items	Alpha	Total (*n* = 734)		Females (*n* = 436)		Males (*n* = 298)		*p* value	Cohen’s *d*
			Mean	SD	Mean	SD	Mean	SD		
*Novelty seeking (NS)*	40	0.75	18.98	5.69	18.87	5.61	19.15	5.81	0.516	0.049
Exploratory excitability (NS1)	11	0.56	6.01	2.28	5.85	2.25	6.24	2.32	0.024	0.170
Impulsiveness (NS2)	10	0.60	3.93	2.16	3.90	2.16	3.99	2.17	0.581	0.041
Extravagance (NS3)	9	0.65	5.03	2.05	5.08	2.09	4.96	2.00	0.422	0.058
Disorderliness (NS4)	10	0.40	4.01	1.89	4.04	1.85	3.97	1.95	0.598	0.036
*Harm avoidance (HA)*	35	0.83	15.15	6.20	16.44	6.21	13.26	5.68	<0.001	0.534
Anticipatory worry (HA1)	11	0.64	4.45	2.39	4.86	2.44	3.85	2.18	<0.001	0.436
Fear of uncertainty (HA2)	7	0.57	4.55	1.68	4.86	1.62	4.11	1.66	<0.001	0.457
Shyness with strangers (HA3)	8	0.66	2.88	2.03	3.16	2.09	2.47	1.88	<0.001	0.347
Fatigability and asthenia (HA4)	9	0.66	3.27	2.18	3.57	2.23	2.83	2.02	<0.001	0.347
*Reward dependence (RD)*	24	0.62	15.44	3.49	15.92	3.44	14.74	3.44	<0.001	0.343
Sentimentality (RD1)	10	0.53	7.10	1.91	7.42	1.82	6.64	1.95	<0.001	0.413
Attachment (RD2)	8	0.64	5.15	2.00	5.15	2.04	5.16	1.93	0.936	0.005
Dependence (RD3)	6	0.48	3.18	1.50	3.35	1.51	2.94	1.44	<0.001	0.277
*Persistence (PS)*	8	0.51	4.44	1.85	4.46	1.90	4.41	1.78	0.699	0.027
*Self-directedness (SD)*	44	0.83	31.19	6.75	30.17	6.75	32.68	6.47	<0.001	0.379
Responsibility (SD1)	8	0.67	5.63	1.94	5.28	2.04	6.15	1.66	<0.001	0.467
Purposefulness (SD2)	8	0.46	5.71	1.59	5.49	1.62	6.03	1.49	<0.001	0.346
Resourcefulness (SD3)	5	0.60	3.49	1.40	3.25	1.45	3.84	1.25	<0.001	0.435
Self-acceptance (SD4)	11	0.72	7.07	2.61	6.85	2.58	7.39	2.63	0.005	0.207
Congruent second nature (SD5)	12	0.57	9.28	2.06	9.30	2.05	9.26	2.07	0.797	0.019
*Cooperativeness (CO)*	42	0.80	32.04	5.51	32.02	5.53	32.06	5.49	0.913	0.007
Social acceptance (CO1)	8	0.62	6.41	1.61	6.38	1.62	6.47	1.60	0.467	0.055
Empathy (CO2)	7	0.38	4.97	1.41	5.05	1.40	4.85	1.42	0.057	0.141
Helpfulness (CO3)	8	0.34	5.92	1.32	5.98	1.32	5.84	1.32	0.151	0.106
Compassion (CO4)	10	0.79	7.90	2.36	7.77	2.36	8.10	2.34	0.063	0.140
Integrated conscience (CO5)	9	0.40	6.83	1.48	6.84	1.50	6.82	1.44	0.813	0.013
*Self-transcendence (ST)*	33	0.81	15.57	5.90	15.94	5.72	15.03	6.11	0.041	0.153
Self-forgetfulness (ST1)	11	0.65	4.92	2.35	4.98	2.34	4.82	2.36	0.374	0.068
Transpersonal identity (ST2)	9	0.70	4.29	2.36	4.17	2.27	4.48	2.47	0.081	0.130
Spiritual acceptance (ST3)	13	0.71	6.36	2.96	6.79	2.88	5.73	2.97	<0.001	0.362

**Table 4:** Principal component analysis of temperament subscales (promax rotation)

	I.	II.	III.	IV.
Novelty seeking (NS)				
Exploratory excitability (NS1)	*−0.47*	*0.40*	0.22	0.03
Impulsiveness (NS2)	0.00	*0.76*	−0.19	−0.14
Extravagance (NS3)	0.03	*0.66*	0.02	0.20
Disorderliness (NS4)	0.14	*0.79*	−0.09	−0.24
Harm avoidance (HA)				
Anticipatory worry (HA1)	*0.83*	0.16	0.27	−0.12
Fear of uncertainty (HA2)	*0.69*	−0.05	0.31	0.11
Shyness with strangers (HA3)	*0.68*	−0.06	−0.09	0.09
Fatigability and asthenia (HA4)	*0.79*	0.16	−0.06	0.01
Reward dependence (RD)				
Sentimentality (RD1)	0.25	−0.05	*0.76*	0.08
Attachment (RD3)	−0.21	0.20	0.29	*0.55*
Dependence (RD4)	0.11	−0.24	−0.14	*0.84*
Persistence (PS)	−0.14	−0.33	*0.63*	−0.32
Explained variance (%)	22.51	16.11	11.61	8.33
Explained total variance (%)	58.56			

Loadings with absolute values of 0.4 or more are shown in italics

**Table 5:** Principal component analysis of character subscales (promax rotation)

	I.	II.	III.
Self-directedness (SD)			
Responsibility (SD1)	*0.70*	0.06	−0.22
Purposefulness (SD2)	*0.84*	−0.08	0.05
Resourcefulness (SD3)	*0.90*	−0.18	0.13
Self-acceptance (SD4)	0.31	0.27	−0.29
Congruent second nature (SD5)	*0.55*	0.22	0.03
Cooperativeness (CO)			
Social acceptance (CO1)	0.11	*0.65*	0.08
Empathy (CO2)	0.11	*0.53*	0.17
Helpfulness (CO3)	0.00	*0.68*	0.00
Compassion (CO4)	−0.09	*0.76*	0.06
Integrated conscience (CO5)	−0.17	*0.69*	−0.11
Self-transcendence (ST)			
Self-forgetfulness (ST1)	−0.04	−0.15	*0.77*
Transpersonal identity (ST2)	0.09	0.16	*0.77*
Spiritual acceptance (ST3)	−0.01	0.12	*0.73*
Explained variance (%)	26.75	14.99	10.50
Explained total variance (%)	52.24		

Loadings with absolute values of 0.4 or more are shown in italics

The correlation matrix between TCI scales and age is shown in Table 6, while the correlation between TCI scales and STAI-S, STAI-T and CES-D is shown in Table 7. The TCI scales correlate significantly but weakly between each other and with age.

**Table 6:** Correlations among TCI scales and age

	NS	HA	RD	PS	SD	CO	ST
Novelty seeking (NS)							
Harm avoidance (HA)	−0.21**						
Reward dependence (RD)	0.11**	0.05					
Persistence (PS)	−0.21**	−0.10**	0.04				
Self-directedness (SD)	−0.10**	−0.57**	0.03	0.08*			
Cooperativeness (CO)	−0.07	−0.20**	0.45**	0.10**	0.42**		
Self-transcendence (ST)	0.10**	−0.06	0.16**	0.23**	−0.20**	0.04	
Age	−0.21**	0.02	0.04	0.09*	0.06	0.09*	0.09*

** *p* < 0.01; * *p* < 0.05

**Table 7:** Correlations among TCI scales and STAI-S, STAI-T and CES-D

	STAI-S	STAI-T	CES-D
Novelty seeking (NS)	0.05	0.01	−0.04
Harm avoidance (HA)	0.31**	0.43**	0.40**
Reward dependence (RD)	0.00	0.01	−0.04
Persistence (PS)	0.01	0.06	0.07
Self-directedness (SD)	−0.35**	−0.47**	−0.48**
Cooperativeness (CO)	−0.16**	−0.19**	−0.23**
Self-transcendence (ST)	0.05	0.05	0.17**

** *p* < 0.01; * *p* < 0.05

## Discussion

The overall psychometric properties of the Greek version of the TCI proved to be satisfactory, with acceptable consistencies of the subscales. Although the study sample was not determined according to strict epidemiological standards and was convenient, it proved to be representative of the general Greek population to a significant extent, especially concerning the healthy, working part of it (it should be noted that the data were gathered before the emergence of the current economic crisis).

The Greek version of the TCI, like its original English counterpart, has good internal consistency. While the current study reports alpha coefficients 0.51–0.83, the Brazilian version has alpha coefficients 0.71–0.87 [33] the Spanish 0.67–0.86 [34], the Finnish 0.61–0.74 [35], the Australian 0.73–0.91 [36], the French 0.49–0.87 [37, 38], the Turkish 0.60–0.85 [39] and the Korean 0.60–0.85 [40].

Overall, the findings, including gender differences, are consistent with the literature. Several individual items proved to have weak psychometric properties. In general, these items were identified by other national studies also and might represent cultural differences, at least partially. The Greek version chose to keep all of them to retain compatibility with other national versions. Also, our results are in line with theoretical considerations as well as with empirical evidence that stems from studies which employed diagnostic interviews for assessing temperament, while the translated questionnaire fulfills criteria for internal validity. Finally, test–retest reliability was not undertaken; however, this is not a serious limitation given the fact that such data exist for TCI from other cultures. It has been reported that TCI manifests a high stability across all factors (ICC = 0.66–0.82) [41].

The TCI [42, 43] was developed to operationalize Cloninger’s personality model [32, 44, 45] and measure the quantitative variation of each of the temperament and character dimensions. It is a 240-item self-report true/false paper-and-pencil questionnaire that takes about 30 min to complete. The test assesses all seven dimensions of personality as previously described. For each dimension, a number of subscales are elaborated to measure facets of the main trait. Extensive data on the reliability and validity of the TCI in the English language have been reported, and the TCI has been shown to have sound psychometric characteristics. In our non-clinical sample, the Greek version of the TCI proved to have sufficient reliability and good validity. It would be relevant to test the TCI in a larger sample of Greek non-clinical subjects, as well as in those with affective disorders, and to look out for relations between temperaments as assessed with TCI and mental disorders at large.

A number of studies have supported the validity of Cloninger’s model and its utility for understanding behavior and clinical syndromes. Cloninger’s model of personality has the potential to provide comprehensive insight into human personality at multiple levels of analysis, including the genetics of personality, the neurobiological foundations of behavior, the cognitive emotional structure and development of personality, the behavioral correlates of individual differences in personality dimensions, and the interactions of personality constellations with developmental factors in relation to the susceptibility to psychiatric disorders. Twin studies and other genetic studies have demonstrated that temperament is moderately heritable, genetically homogeneous and probably individual temperaments are independent of one another [46–49]. On the contrary, character is weakly heritable, and moderately influenced by family environment and social learning. Character development can be operationalized in terms of the abstract symbolic processes that are most highly evolved in humans, such as voluntary goal-directed behavior, empathic social cooperation, and creative symbolic invention [50]. There are some data suggesting that this model is related to aspects of DSM personality disorders especially concerning Self-directedness and Cooperativeness [29, 51–56] or with characteristics of mood disorders [57, 58] and with other mental disorders [59, 60]. Other dimensions (e.g., novelty seeking, harm avoidance) have shown to relate with various clinical syndromes such as depression, anxiety and alcoholism.

Although the description of temperaments and characters is out of the scope of the current paper, briefly it should be mentioned that NS encompasses bias in the activation of behavior such as exploratory activity in response to novelty, impulsive decision making, extravagance in the approach to cues of reward, and quick loss of temper. HA consists of bias in the inhibition or cessation of behaviors, such as pessimistic worry in anticipation of future problems, passive avoidant behaviors such as fear of uncertainty and shyness of strangers, and rapid fatigability. RD includes bias in the maintenance of ongoing behaviors, and is exhibited as sentimentality, social attachment, and dependence on the approval of others. PS is manifest as a bias in resistance to extinction of behavior when confronted with frustrative lack of reward. SD quantifies the extent to which an individual is responsible, purposeful, resourceful, self-accepting, and dutiful. CO is the degree to which a person identifies with other people and feels like an integral part of society; cooperative people are tolerant, empathic, helpful, compassionate, and principled. ST measures the extent to which a person feels like an integral part of the universe as a whole; self-transcendent people are imaginative, self-forgetful, faithful, spiritual, and idealistic.

Validation studies for TCI translations include a Serbian [61], Spanish [62], Swedish [63], Brazilian [33], Finnish 0.61–0.74 [35], Australian [36], French [37, 38, 64], Turkish [39], and Korean [40] versions among others. Their results are more or less in accord with the results reported by the current study.

The limitations of the current study include the fact that the study sample was not typically representative of the general population of the country and the selection method was not standardized. A second limitation is the lack of reporting of correlations with similar tests already validated in Greece.

## Conclusion

The current validation study of the Greek version of the TCI reported that this version has psychometric properties similar to other national translations and to the original scale, and it is suitable for use in research and clinical practice.
